# Impact of economic growth target constraints on enterprise technological innovation: Evidence from China

**DOI:** 10.1371/journal.pone.0272003

**Published:** 2022-08-02

**Authors:** Enji Li, Ziwei An, Chen Zhang, Hua Li

**Affiliations:** 1 School of Business Administration, Hebei University of Economics and Business, Shijiazhuang, Hebei, China; 2 Institute of Social Governance, Hebei University of Economics and Business, Shijiazhuang, Hebei, China; 3 School of Public Finance & Taxation, Shandong University of Finance and Economics, Jinan, Shandong, China; 4 School of Business Administration, University of Science and Technology Liaoning, Anshan, Liaoning, China; Ball State University, UNITED STATES

## Abstract

By using the data from Chinese listed companies in the Shanghai and Shenzhen A-share market for the period 2007 to 2019 and the *Report on the Work of the Government*, this paper investigates the impact of the local government’s economic growth target constraints on enterprise technological innovation and its mechanism. It is found that the local government’s economic growth target constraints significantly inhibit enterprise technological innovation, which is more obvious in the samples of SOEs, regulated industries, and enterprises at low marketization levels. The change of government officials’ performance appraisal indicators from the quantity to quality of economic growth can alleviate the negative effect. The mechanism effect test indicates that the local government’s economic growth target constraints will aggravate enterprise financing constraints and decrease the contribution of R&D investment to enterprise performance, and further inhibit enterprise technological innovation.

## I. Introduction

Enterprises are the main body of the technological innovation system and the improvement of its innovation ability is the guarantee of national innovation quality and efficiency. However, due to the characteristics of high investment, long cycle, and high risk of technological innovation activities, and the property of quasi-public goods of R&D achievements, enterprises cannot obtain the full benefits of innovation, leading to the enterprise R&D investment being far lower than the ideal level [1, 2]. In this instance, it is necessary for governments to implement regulations, correct market failures, and motivate enterprise technological innovation enthusiasm. In a sense, enterprise technological innovation is not only the result of market choice but also the government promotion [3].

The management of economic growth targets is one of the most important ways for the government to regulate macro-economic development and has a major impact on regional resource allocation. In China, there triggers an intensive tournament competition in local government’s fiscal expenditure [4, 5] and environmental regulation [6, 7] because of the strong linkage between the private interests of government officials and regional economic development [8, 9]. The tournaments also exit in local government’s economic growth target setting, mainly showing that the value of economic growth targets is amplified along with different jurisdiction levels [10, 11]. The relevant studies have shown that economic growth target setting necessarily aroused the local government’s enthusiasm to leverage local investment which successfully promotes the brilliant achievements of China’s economy in the past four decades [12]. However, driven by political targets, economic growth targets may be divorced from reality. Faced with a restrictive assessment indicator, in order to achieve the established goals, local governments tend to adopt short-term economic actions [13], leading to environmental pollution [14], the distortion of fiscal expenditure structure [15], and the reduction of energy efficiency [16], etc. The local government and its officials play an important role in allocating regional innovation resources, and the local government’s behavior is viewed as a important economic indicator that affects enterprise operations, investments, and other activities [11]. Our question is how will the local government’s economic growth target constraints affect the enterprise technological innovation activities? What is its mechanism?

In addition, with China’s economy entering new normal, the tendency to assess government officials’ performance only by GDP growth rate has been weakened, replaced by the indicators reflecting the quality of economic development, such as environmental protection and scientific and technological innovation, which raise another question. As the root of economic growth target constraints changes, will it affect the relationship between economic growth target constraints and enterprise technological innovation? The answers to these questions are important to stimulate enterprise technological innovation vitality, and can offer theoretical reference for local governments to make relative policies.

The previous literature made a profound discussion about the impact of the local government’s economic growth target constraints on technological innovation activities, forming two diametrically opposing ideas. Macrocosmically, some scholars argued that higher economic growth targets have a prominent inhibitory effect on regional technological innovation, and there existed significant heterogeneities with urban characteristics in different stages of development [17, 18]. Different from their conclusions, Wang et al. [19] further distinguished economic growth targets’ hard-constraint and soft-constraint, finding that the former can inhibit regional technological innovation and the latter can facilitate urban technological innovation. Wang et al. [20] showed that the impact of economic growth target constraints on regional technological innovation is inverted U-shaped. From the micro level, Lv and Tao [21] believed that the local government’s economic growth target constraints have promoted enterprise technological innovation. Conversely, Li et al. [22] considered that although the pressure of economic growth targets has a crowding-out effect on enterprise R&D expenditure, it would improve the efficiency of enterprise technological innovation.

Overall, although enterprises are the important microcosmic foundation of high-quality macroeconomic development, the research on the impact of local government’s economic growth target constraints on enterprise technological innovation activities is rather little and debatable. The measurement of the local government’s economic growth targets places emphasis on a certain government level, for example, the margin between the economic growth target of the municipal government and the provincial government is used in [21], and the margin between the provincial government target growth rate and the actual growth rate of the current year is used in [22]. But as the economic growth target is top-down amplified gradually, it is difficult to measure the growth pressure faced by the government and estimate its effects on enterprise technological innovation at a single level of the government’s economic growth target constraints. Moreover, the extant literature mostly focused on the government’s economic growth target setting and its influence under the GDP-oriented evaluation mechanism. However, as the central government gradually strengthens the evaluation of the performance of local governments and officials in scientific and technological innovation, the local officials’ promotion strategies are bound to change, but the extant literature pays less attention to the official performance evaluation reform.

Our paper tries to explore the impact of economic growth target constraints on the enterprise technological innovation by matching the data of Chinese listed companies in the Shanghai and Shenzhen Stock A-share Market with provincial and urban panel data from 2007 to 2019. Our study complements the extant literature in three aspects. Firstly, different from extant research that only focuses on the economic growth target constraints of local governments at a certain level, we use three ways to measure the economic growth target constraints of local governments at different levels, which are more in line with the setting process of local government’s economic growth target and the formation of local government’s economic growth target constraints. We also compare the heterogeneous effects of economic growth target constraints of local governments at different levels, which can offer a better comprehension about enterprise technological innovation activities from the perspective of the local government’s economic growth target management. Secondly, we test the mechanism of local government’s economic growth target constraints on enterprise technological innovation from two aspects of financing constraints and the contribution of R&D investment to enterprise performance, which build a new logical bridge between macroeconomic management and micro-enterprise technological innovation, and are helpful for open the mechanism “black box” of the impact of economic growth target constraints on enterprise technological innovation. Thirdly, we evaluate the moderating effect of the reform of government officials’ performance appraisal on the relationship between the economic growth target constraints and the enterprise technological innovation activities, contributing to a better understanding of the government-level factors in the enterprise technological innovation activities, and providing theoretical support for optimizing government officials’ performance appraisal system.

The remainder of the paper is structured as follows. Section II sets out the institutional backgrounds and hypothesis development. Section III describes the research design and Section IV reports the empirical results of our hypothesis. Section V estimates the heterogeneous influence and mechanism of local government’s economic growth target constraints on enterprise technological innovation. Section VI concludes the paper and discusses the policy implications.

## II. Institutional backgrounds and hypothesis development

Economic growth is, without doubt, one of the core tasks faced by local governments in China, and the economic growth target plays a predominant role in the government agenda from the period of planned economy to market economy. Setting the economic growth target is a top-down process, the central government, provincial governments, and municipal governments, in turn, set up the annual national, provincial, and city growth targets. It usually takes several months and several rounds of consultation before annual economic growth targets are released in the *Report on the Work of the Government* [11, 23].

Under the Sinicism decentralization and evaluation mechanism that emphasizes relative performance, the value of economic growth targets is top-down amplified along with different jurisdiction levels. After the central government proposes the annual economic growth targets, by changing targets and affecting the promotion rules of subordinates, provincial governments convey to municipal governments the importance of economic growth in their job responsibilities and require municipal governments to provide higher economic growth targets to strive for better economic performance [9, 24]. The same pattern is followed when municipal governments direct county governments to set economic growth targets. For subordinate governments, in order to send an "ability signal" to the superior government and get promoted, a proper target should both in line with their past goal levels and attainment discrepancies, horizontally targeting the comparable peers’ goal levels to guarantee their targets are higher than their competitors, and vertically aligning with the upper-tier authorities’ mandates [10, 25]. Meanwhile, when announcing annual growth targets, local governments tend to use "ensure" and "above" to declare themselves and win the yardstick competition at the comparable peers’ levels [26].

Under the pressure of economic growth, local governments try their best to obtain beneficial factor resources for economic growth by attracting investment, building infrastructure, and providing preferential policies and other measures, which play a vital role in China’s rapid economic growth [27–30]. However, when local governments anticipate that they will fail to reach the promised economic growth targets according to the market rules, radical behavior to strive for short-term targets will appear, which has a series of adverse effects on enterprise technological innovation environment and the acquisition of innovation resources [31–33].

First of all, under the restraint of economic growth targets, local governments prefer to invest in infrastructure and reduce the innovation investments of "long investment cycle, high risk" in order to rapidly boost the GDP of the jurisdiction, and then the fiscal science and technology expenditure is reduced passively, destroying the enterprise technological innovation environment. Secondly, in order to achieve the established growth targets, local governments will use their administrative power to transfer the growth targets to enterprises, especially the state-owned enterprises, which consume the limited resources of enterprises and squeeze out the enterprise technological innovation resources. The political-ecological environment is vital for enterprises, to cater to the needs of the government for short-term investment and establish a good relationship with the government, enterprises will also actively reduce the long-term investment including the R&D investment, and increase short-term investment [34–36]. Thirdly, when the economic growth pressure increases, the economic growth target constraints will further strengthen governments’ investment impulse and enlarge the scale of governments’ debt, which will reduce the efficiency of jurisdiction credit allocation, worsen the financing constraints of the private sector and adversely affect enterprise technological innovation [37]. Finally, to pursue economic performance, local governments’ intervention and control of land, capital, labor, and other factors may also be strengthened, distorting the allocation of market resources, which in turn increases enterprise technological innovation costs. Accordingly, we state our first hypothesis:

**Hypothesis 1:** Local government’s economic growth target constraints negatively affect enterprise technological innovation.

In recent years, the central government constantly issued a series of standard documents to reform and optimize the government officials’ performance appraisal system. At the *National Organization Work Conference* in 2013, General Secretary Xi Jinping pointed out that the government officials’ performance appraisal can no longer be judged simply by the GDP growth rate. At the end of 2013, the *Organization Department of the CPC Central Committee* issued a *Notice on Improving the Performance Assessment of Local Party and Government Leaders Officials* stressing that scientific and technological innovation will become essential for the government officials’ assessment. Under the background, the weight of economic growth indicators has declined, the pursuit of short-term economic growth by local governments has been weakened, and the amplification of growth targets becomes much smaller than previous [11]. Taking 2012 as the demarcation point, the average differences of economic growth targets between the municipal and provincial governments, and the municipal governments and the central government were 2.4% and 5.1% during 2007–2012, respectively, but they decreased to 0.6% and 2.2% in 2013–2018. Meanwhile, the transformation of the official performance evaluation system has provided local governments with the motivation to "compete for innovation", technological innovation has also gradually become a new competition criterion in official tournaments, and the attitude of local governments towards scientific and technological innovation has changed significantly. In order to steal a march on the new round of competition, local governments have increased their investment and support for technological innovation activities. On the one hand, local governments have expanded fiscal science and technology expenditure, increased innovation subsidies or strengthened tax preferential policies, and eased the financing constraints of innovation subjects. It is found that the local government’s scientific and technological expenditure has increased obviously after 2013, and there is increasingly heated competition among local governments over scientific and technological expenditure [38]. On the other hand, local governments are committed to guiding the social production factors rationally flow to the R&D sector through various directives, policies, and plans, so as to create a favorable soft environment for technological innovation activities. In light of the above argument, our second hypothesis is stated as follows:

**Hypothesis 2:** The negative effect of the local government’s economic growth target constraints on enterprise technological innovation can be alleviated by the reform of official performance evaluation.

## III. Research design

### 1. Sample and data selection

The data of the micro-enterprise and the macro government’s economic growth targets are used in our paper. Microenterprise data are from Chinese listed companies in the Shanghai and Shen Zhen A-share market between 2007 and 2019. We eliminate the sample including the companies that went through ST, ST* and delisted companies during the sample period; financial listed companies; the companies’ R&D expending and patent data are severely missing during the sample period; the companies with abnormal financial data, like negative total assets, total assets less than current assets or intangible assets, and negative liabilities. The patent data come from the website of the State Intellectual Property Office of China, other enterprise data come from the China Stock Market and Accounting Research (CSMAR) database and the China Center for Economic Research (CCER) database.

The data about the government’s economic growth targets are collected by hand from the central, provincial, and municipal government reports over the years. The sample period of the central and provincial governments’ economic growth targets is from 2007 to 2019, and the municipal government’s economic growth targets are from 2007 to 2018. It is worth noting that, when the economic growth target is expressed by the “interval”, the mean value is used as the government’s economic growth target. For example, when the proposed economic growth target is 5% to 5.5%, we use 5.25% as the government’s economic growth target.

The final sample consists of 2246 firm-year observations. In order to eliminate the effect of extreme values and increase the reliabilities of the conclusion, we winsorize enterprise variables at the 1 and 99 percentiles (see **S1 Dataset** for details).

### 2. Model and variables

The following model is constructed to investigate the impact of the local government’s economic growth target constraints on enterprise innovation:

$${\mathrm{Innovation}}_{i,t}=\alpha_{0}+\alpha_{1}\mathrm{Constraints}+\overset{n}{\underset{j=2}{\sum }}\alpha_{j}X_{i,t}+\mu_{i}+\lambda_{t}+\nu_{it}$$ (1)

The dependent variable is enterprise technological innovation (*Innovation*_*i*,*t*_). Referring to the extant literature, we use R&D input and the invention patent applications together to measure enterprise technological innovation. Enterprise technological innovation input (*R&D*) is defined as the natural logarithm of 1 plus the R&D expenditure. In the robustness test, the proportion of enterprise R&D investment in operating income (*R&D ratio*) is used to measure enterprise technological innovation input. For the output index of enterprise innovation, according to the relevant provisions of the *Implementing Regulations of the Patent Law of the People’s Republic of China*, patents include the invention patent, utility models, and designs, of which the invention patent is considered to have the highest technical content and can better reflect the level of enterprise technological innovation. So, we define enterprise technological innovation output as the natural logarithm of 1 plus the annual invention patent applications (*Patent*).

The core explanatory variable is the economic growth target constraints of local governments (*Constraints*_*c*,*t*_). According to Yu and Pan [25], the provincial government’s economic growth target constraints are defined as the difference of the economic growth target between provincial governments and the central government (*Constraint1*), the municipal government’s economic growth target constraints are defined as the difference of the economic growth target between the municipal government and the provincial government (*Constraint2*). The difference of the economic growth target between the municipal government and the central government is the cumulative results of multi-level government competition [11, 24], and we define it as *Constraint3*.

Following the relevant research [39, 40], *X*_*i*,*t*_ is a set of control variables of enterprise, including enterprise size (*Size*), the return on assets (*ROA*), enterprise age (*Age*), the percentage of shares owned by the top5 largest shareholder (*Top5*), the amount of enterprise staff (*Staff*), enterprise solvency (*Lev*), enterprise growth (*Tobin Q*), and enterprise ownership *(Dum_stat*e);. *μ_i_* represents industry fixed effect, *λ_t_* represents time fixed effect, and *ν_t_* is random error.

## IV. Empirical results and analysis

### 1. Descriptive statistics

**Table 1** presents descriptive statistics for major variables. The mean of the R&D expenditure (*R&D*) and invention patent applications (*Patent*) are about 37.13 million (the log value is about 17.430) and 7 pieces (the log value is about 1.936) and the standard deviations are 2.145 and 1.323, respectively, indicating that there exists a significant difference in the enterprise technological innovation. Local governments at all levels confront different degrees of economic growth target constraints. The mean of the provincial government’s economic growth target constraints (*constraint1*) is about 1.13%, meaning that the provincial government’s economic growth targets exceed the central government by an average of about 1.13 percentage points. The mean of the municipal government’s economic growth target constraints (*constraint2*) is about 0.96%, meaning that the municipal government’s economic growth targets exceed the provincial government by an average of about 0.96 percentage points. In view of the amplification of the provincial government’s economic growth targets, the municipal government’s economic growth target is about 2.25% higher than the central government’s economic growth target.

**Table 1:** Descriptive statistics of major variables.

Variable Name	Variable Meaning	Observations	Mean	Std. Dev.	Min	Max
**R&D**	the natural logarithm of 1 plus the R&D expenditure	14797	17.430	2.145	4.185	21.570
**Patent**	the natural logarithm of 1 plus the invention patent applications	11043	1.936	1.323	0	5.964
**Constraint1**	the difference of the economic growth target between the provincial governments and the central government(%)	14797	1.137	1.181	-1.750	7
**Constraint2**	the difference of the economic growth target between the municipal government and the provincial government (%)	11530	0.962	1.214	-5	15
**Constraint3**	the difference of the economic growth target between the municipal government and the central government (%)	11530	2.246	1.907	-5.500	18.500
**Size**	the natural logarithm of the enterprise asset	14797	21.930	1.183	17.810	28.190
**Tobin Q**	Tobin Q	14797	2.683	1.815	0.858	11.440
**Lev**	asset-liability ratio(%)	14797	0.399	0.199	0.0542	0.907
**Age**	enterprise age	14797	15.330	5.585	4	30
**Top5**	the percentage of shares owned by the top5 largest shareholder(%)	14797	52.430	14.270	18.930	89.080
**ROA**	the return on assets(%)	14797	0.0361	0.0654	-0.276	0.195
**Staff**	the natural logarithm of the enterprise staff	14797	5.520	1.312	0	10.490
**Dum_state**	The value is 1 if the firm is actually controlled by SOE, otherwise, the value is 0	14797	0.284	0.451	0	1

**Table 2** shows the correlation coefficients of the major variables. The Pearson correlation coefficients of R&D and the invention patent applications with the different local government’s economic growth target constraints are negative and significant at the 1% level, providing a preliminary test for subsequent empirical analysis.

**Table 2:** The correlation coefficient of major variables.

	R&D	Patent	Constraint1	Constraint2	Constraint3	Size	Tobin Q	Lev	Age	ROA
**R&D**	1									
**Patent**	0.395***	1								
**Constraint1**	-0.172***	-0.046***	1							
**Constraint2**	-0.183***	-0.073***	0.287***	1						
**Constraint3**	-0.215***	-0.081***	0.794***	0.810***	1					
**Size**	0.179***	0.354***	-0.088***	-0.099***	-0.113***	1				
**Tobin Q**	-0.068***	-0.073***	-0.042***	-0.051***	-0.070***	-0.429***	1			
**Lev**	0.032***	0.100***	0.076***	0.039***	0.076***	0.452***	-0.340***	1		
**Age**	-0.085***	0.003	-0.188***	-0.167***	-0.188***	0.212***	-0.106***	0.124***	1	
**ROA**	0.012*	0.081***	0.003	0.024***	0.003	-0.014**	0.242***	-0.347***	-0.091***	1

Notes:

***, ** and *represent significance at the 1%, 5%, and 10% levels, respectively.

### 2. The impact of economic growth target constraints on enterprise technological innovation

#### 2.1. Basic model results

**Table 3** reports the regression results of Formula (1), which tests the impact of economic growth target constraints on enterprise technological innovation. Using the R&D expenditure to measure enterprise technological innovation, the regression coefficients of local governments’ economic growth target constraints are -0.083, -0.009, and -0.043, respectively. In addition to *Constraint2* failing the significance test, *Constraint1* and *Constraint3* are significant at the level of 1% and 5%. Using the natural logarithm of enterprise invention patents to measure enterprise technological innovation, the regression coefficients of local governments’ economic growth target constraints are -0.042, -0.031, and -0.033, respectively, which are significant at the level of 5%, 5%, and 1%. These results support H1, that is, the local government’s economic growth target constraints inhibit enterprise technological innovation. According to the regression results of Columns (3) and (6), when the municipal government’s economic growth target is 1% higher than the central government’s, enterprise R&D expenditure and the invention patent applications will decrease by about 4.3% and 3.3%, respectively. From the perspective of enterprise financing constraints, there are two reasons. First, when confronted with economic growth pressure, local governments tend to neglect the quality of economic development, adopt stimulative economic policies dominated by expansive fiscal policy, allocate more resources to infrastructure, and reduce the innovation input with a long investment cycle, which will inhibit the technological innovation activities of enterprises. Second, strict target constraints will result in more severe distortions in credit resources, improving the enterprise financing cost and reducing enterprise technological innovation enthusiasm.

**Table 3:** The impact of economic growth target constraints on enterprise technological innovation.

	(1)	(2)	(3)	(4)	(5)	(6)
	R&D	R&D	R&D	Patent	Patent	Patent
**Constraint1**	-0.083***			-0.042**		
	(0.022)			(0.019)		
**Constraint2**		-0.009			-0.031**	
		(0.024)			(0.015)	
**Constraint3**			-0.043**			-0.033***
			(0.017)			(0.012)
**Size**	0.685***	0.723***	0.720***	0.510***	0.529***	0.528***
	(0.033)	(0.037)	(0.036)	(0.036)	(0.038)	(0.037)
**Tobin Q**	0.047***	0.037**	0.037**	0.066***	0.068***	0.070***
	(0.013)	(0.015)	(0.015)	(0.013)	(0.013)	(0.013)
**Lev**	-0.449***	-0.452***	-0.445***	-0.260*	-0.325**	-0.333**
	(0.140)	(0.165)	(0.162)	(0.140)	(0.148)	(0.145)
**Age**	-0.025***	-0.028***	-0.028***	-0.014***	-0.017***	-0.017***
	(0.005)	(0.006)	(0.006)	(0.005)	(0.005)	(0.005)
**Top5**	0.000	0.000	-0.000	-0.006***	-0.007***	-0.007***
	(0.002)	(0.002)	(0.002)	(0.002)	(0.002)	(0.002)
**ROA**	1.732***	2.402***	2.366***	1.551***	1.573***	1.583***
	(0.317)	(0.398)	(0.394)	(0.409)	(0.428)	(0.422)
**Staff**	0.326***	0.259***	0.257***	0.086***	0.075***	0.074***
	(0.023)	(0.022)	(0.022)	(0.017)	(0.017)	(0.017)
**Dum_state**	-0.061	-0.069	-0.057	0.077	0.077	0.093
	(0.068)	(0.073)	(0.073)	(0.061)	(0.062)	(0.062)
**Industry FE**	Yes	Yes	Yes	Yes	Yes	Yes
**Year FE**	Yes	Yes	Yes	Yes	Yes	Yes
**N**	14797	11529	11731	11043	10020	10205
**F**	191.215	137.634	139.035	44.472	42.111	43.438
**Adjusted R** ^**2**^	0.517	0.515	0.516	0.239	0.243	0.244

Notes: The standard errors are clustered by firm and reported in the parenthesis.

***, ** and *represent significance at the 1%, 5%, and 10% levels, respectively.

Comparing the regression coefficients of *Constraint1*, *Constraint2*, and *Constraint3*, we also find that the local government’s economic growth target constraints from different levels have a distinguishing impact on enterprise technological innovation. The amplification of municipal governments’ targets on the provincial governments’ targets has a limited impact on the enterprise technological innovation activities, and the amplification of provincial governments’ targets on the central government’s targets plays the dominant role. That is to say, the competition between the provincial governments around the setting of economic targets has a more profound impact on the political-ecological environment where enterprises are located.

Control variables such as enterprise size (*Size*), enterprise growth (*Tobin Q*), enterprise *ROA*, and the amount of enterprise staff (*Staff*) are positive with the enterprise technological innovation while enterprise age (*Age*) and asset-liability rates *(Lev*) are negatively related to the enterprise innovation. These results are largely consistent with the previous literature [20, 39].

### 2.2. Robustness check

*2*.*2*.*1*. *Endogeneity discussion*. In this paper, the government’s economic growth target is exogenous relative to a single enterprise, but since the government’s basis to set setting economic growth target is the overall performance of the enterprises in the jurisdiction, there may still be an inverse causal relationship in the model. At the same time, although a series of control variables are added to the model, there may still have other factors affecting both the enterprise technological innovation and the local government’s economic growth targets setting, and if they are placed in the random error, the estimated results will be biased. To alleviate the endogenous problem, we carry out the following methods. Firstly, referring to Li et al. [22], we use two-period and three-period lag of the local government’s economic growth target constraints as instrumental variables and use two stages least squares (2SLS) to re-estimate. The estimation results of the first stage show that the two-period and three-period lag of the local government’s economic growth target constraints are significantly related to the current local government’s economic growth target constraints. The estimated results of the second stage are shown in Columns (1) to (3) of **Table 4**. In all regressions, the Kleibergen-Paap rk LM test rejects the null hypothesis that the instrumental variables and the endogenous explanatory variable are not related, the Cragg-Donald Wald F-value is significantly higher than the critical value of the weak instrumental variable test, and the Hansen test cannot reject the null hypothesis that the instrumental variables are exogenous, so it can be assumed that the instrumental variables are reasonable. The regression coefficients of *Constraint1*, *Constraint2*, and *Constraint3* are obviously negative, which is consistent with the previous conclusions. Secondly, in order to further alleviate endogeneity problems caused by omitted variables, we use the lag term of dependent variables (*L*.*Patent*) as instrumental variable and use the system generalized method of moments (GMM) to re-estimate. The results are shown in **Columns (4)-(6) of Table 4**, the P-value of AR (2) shows that there is no second-order sequence correlation of the disturbance term, and the Hansen test shows that the model does not have an overidentifying problem, showing that our estimate is effective. The regression results all passed the Arellano-Bond test and the Hansen test. The estimated coefficient of the economic growth target constraints is significantly negative at the 5% confidence level when the independent variables are *Constraint1* and *Constraint3*. While the estimated coefficient of *Constraint2* is negative, not significant, which is a little bit different from the benchmark results. Overall, H1 is confirmed and the result is convinced largely.

**Table 4:** The estimated results of IV-2SLS and GMM.

	(1)	(2)	(3)	(4)	(5)	(6)
**Constraint1**	-0.073***			-0.055**		
	(0.022)			(0.023)		
**Constraint2**		-0.073**			-0.028	
		(0.029)			(0.025)	
**Constraint3**			-0.053***			-0.033**
			(0.015)			(0.016)
**Size**	0.526***	0.557***	0.553***	0.474***	0.457***	0.469***
	(0.024)	(0.027)	(0.026)	(0.055)	(0.057)	(0.056)
**Tobin Q**	0.058***	0.060***	0.060***	0.123***	0.114***	0.118***
	(0.011)	(0.012)	(0.012)	(0.024)	(0.025)	(0.024)
**Lev**	-0.383***	-0.508***	-0.521***	-0.317	-0.288	-0.314
	(0.108)	(0.118)	(0.116)	(0.204)	(0.211)	(0.205)
**Age**	-0.018***	-0.026***	-0.025***	-0.011**	-0.015***	-0.015***
	(0.004)	(0.004)	(0.004)	(0.005)	(0.005)	(0.005)
**Top5**	-0.009***	-0.011***	-0.011***	-0.008***	-0.008***	-0.009***
	(0.001)	(0.001)	(0.001)	(0.002)	(0.002)	(0.002)
**ROA**	1.300***	1.551***	1.571***	1.005	1.209	1.161
	(0.332)	(0.358)	(0.354)	(0.910)	(0.918)	(0.902)
**Staff**	0.136***	0.109***	0.108***	0.067***	0.046**	0.045*
	(0.014)	(0.015)	(0.015)	(0.024)	(0.023)	(0.023)
**Dum_state**	0.097**	0.102**	0.126***	-0.042	-0.033	-0.020
	(0.039)	(0.041)	(0.041)	(0.067)	(0.066)	(0.066)
**Industry FE**	Yes	Yes	Yes	Yes	Yes	Yes
**Year FE**	Yes	Yes	Yes	Yes	Yes	Yes
**Kleibergen-Paap rk LM statistic (p value)**	1146.454	350.196	672.774			
	(0.000)	(0.000)	(0.000)			
**Cragg-Donald Wald F statistic**	2931.800	1251.302	2539.088			
**Hansen J statistic**	2.492	0.033	0.890			
**(p value)**	(0.114)	(0.855)	(0.3454)			
**AR(1)**				0.000	0.000	0.000
**AR(2)**				0.503	0.181	0.162
**Hansen test**				0.359	0.190	0.176
N	6278	5328	5435	8487	7249	7372

Notes: The dependent variable is enterprise innovation patent (*Patent*) in all regressions. The standard errors are clustered by firm and reported in the parenthesis.

***, ** and *represent significance at the 1%, 5%, and 10% levels, respectively.

Finally, the model formalization error can also make estimated results biased. For this, we adopt the debiased machine learning method developed by Chernozhukov et al. [41], relaxing the linear hypothesis for estimation. In the regression, we add all control variables in Eq (1), the estimated results are shown in **Table 5**, which are consistent with the benchmark, too.

**Table 5:** The regression results of debiased machine learning.

	(1)	(2)	(3)	(4)	(5)	(6)
	R&D	R&D	R&D	Patent	Patent	Patent
**Constraint1**	-0.086***			-0.042***		
	(0.014)			(0.010)		
**Constraint2**		-0.009			-0.032***	
		(0.017)			(0.009)	
**Constraint3**			-0.044***			-0.033***
			(0.012)			(0.007)
**Control variables**	Yes	Yes	Yes	Yes	Yes	Yes
**Industry FE**	Yes	Yes	Yes	Yes	Yes	Yes
**Year FE**	Yes	Yes	Yes	Yes	Yes	Yes
**Wald chi2(1)**	37.33	0.28	14.04	16.58	11.54	25.17
**N**	14797	11529	11731	11043	10020	10205

Notes: The robust standard errors are reported in the parenthesis.

***, ** and *represent significance at the 1%, 5%, and 10% levels, respectively.

*2*.*2*.*2*. *Other robustness tests*. Since the measurement of dependent variables and estimation methods may influence the conclusion, firstly, we use the strength of enterprise R&D (*R&D ratio* is defined as R&D expenditure divided by enterprise operating income) to re-measure enterprise technological innovation and the regression results are shown in Columns (1) to (3) of **Table 6**. Secondly, based on the number of invention patent applications, we generate a dummy variable (*Dummy_invention*). If the number of invention patent applications is greater than the sample average, the *Dummy_invention* value is 1, otherwise, the *Dummy_invention* value is 0. We use *Dummy_invention* as the explanatory variable and use the logit model to re-regress. The estimated results of average marginal effect are shown in Columns (4) to (6) of **Table 6**, and are generally consistent with the benchmark results.

**Table 6:** Other robustness tests: Replacing the measurement of the explained variable and the estimation methods.

	(1)	(2)	(3)	(4)	(5)	(6)
	R&D ratio			The original value of the invention patents		
**Constraint1**	-0.001**			-0.274***		
	(0.001)			(0.016)		
**Constraint2**		-0.001*			-0.104***	
		(0.000)			(0.016)	
**Constraint3**			-0.001***			-0.117***
			(0.000)			(0.011)
**Size**	0.279***	0.376***	0.354***	0.279***	0.376***	0.354***
	(0.019)	(0.022)	(0.022)	(0.019)	(0.022)	(0.022)
**Tobin Q**	-0.065***	-0.022*	-0.030**	-0.065***	-0.022*	-0.030**
	(0.011)	(0.013)	(0.013)	(0.011)	(0.013)	(0.013)
**Lev**	-0.157	-0.327***	-0.252**	-0.157	-0.327***	-0.252**
	(0.108)	(0.122)	(0.122)	(0.108)	(0.122)	(0.122)
**Age**	0.061***	0.036***	0.032***	0.061***	0.036***	0.032***
	(0.003)	(0.004)	(0.004)	(0.003)	(0.004)	(0.004)
**Top5**	0.001	0.001	-0.000	0.001	0.001	-0.000
	(0.001)	(0.001)	(0.001)	(0.001)	(0.001)	(0.001)
**ROA**	-1.523***	-1.224***	-1.061***	-1.523***	-1.224***	-1.061***
	(0.276)	(0.348)	(0.344)	(0.276)	(0.348)	(0.344)
**Staff**	0.013	0.008	0.008	0.013	0.008	0.008
	(0.013)	(0.014)	(0.014)	(0.013)	(0.014)	(0.014)
**Dum_state**	-0.253***	-0.241***	-0.174***	-0.253***	-0.241***	-0.174***
	(0.040)	(0.044)	(0.044)	(0.040)	(0.044)	(0.044)
**Industry FE**	Yes	Yes	Yes	Yes	Yes	Yes
**Year FE**	Yes	Yes	Yes	No	No	No
**N**	14796	11528	11730	16621	13262	13484
**F**	61.504	48.370	49.361			
**Adjusted R** ^**2**^	0.364	0.360	0.358			
**Pseudo R2**				0.0809	0.0511	0.0560

Notes: The standard errors are clustered by firm and reported in the parenthesis.

***, ** and *represent significance at the 1%, 5%, and 10% levels, respectively.

In addition, considering the impact of the economic growth target constraints on enterprise technological innovation may appear in later terms, we use the one-period lag of the enterprise technological innovation as the dependent variable. The results are shown in **Table 7**, supporting the conclusion mentioned above too.

**Table 7:** Other robustness tests: The enterprise technological innovation lags by one period.

	(1)	(2)	(3)	(4)	(5)	(6)
	F.R&D	F.R&D	F.R&D	F.Patent	F.Patent	F.Patent
**Constraint1**	-0.088***			-0.049**		
	(0.020)			(0.019)		
**Constraint2**		-0.012			-0.034**	
		(0.020)			(0.017)	
**Constraint3**			-0.043***			-0.036***
			(0.015)			(0.012)
**Size**	0.679***	0.719***	0.717***	0.527***	0.548***	0.546***
	(0.030)	(0.034)	(0.033)	(0.038)	(0.041)	(0.040)
**Tobin Q**	0.059***	0.062***	0.061***	0.080***	0.080***	0.081***
	(0.011)	(0.013)	(0.013)	(0.013)	(0.014)	(0.014)
**Lev**	-0.244*	-0.288*	-0.285*	-0.107	-0.182	-0.188
	(0.133)	(0.150)	(0.148)	(0.156)	(0.165)	(0.162)
**Age**	-0.023***	-0.027***	-0.027***	-0.015***	-0.018***	-0.018***
	(0.005)	(0.005)	(0.005)	(0.005)	(0.006)	(0.006)
**Top5**	0.000	0.000	-0.000	-0.006***	-0.006***	-0.007***
	(0.002)	(0.002)	(0.002)	(0.002)	(0.002)	(0.002)
**ROA**	2.796***	3.275***	3.238***	2.401***	2.523***	2.524***
	(0.325)	(0.379)	(0.375)	(0.440)	(0.464)	(0.457)
**Staff**	0.273***	0.244***	0.242***	0.061***	0.053***	0.053***
	(0.021)	(0.022)	(0.022)	(0.018)	(0.019)	(0.019)
**Dum_state**	-0.039	-0.050	-0.039	0.099	0.100	0.119*
	(0.064)	(0.069)	(0.069)	(0.065)	(0.067)	(0.066)
**Industry FE**	Yes	Yes	Yes	Yes	Yes	Yes
**Year FE**	Yes	Yes	Yes	Yes	Yes	Yes
**N**	13776	11571	11778	9234	8416	8575
**F**	203.449	158.017	161.775	42.390	40.415	41.659
**Adjusted R** ^**2**^	0.871	0.397	0.398	0.230	0.237	0.238

Notes: The standard errors are clustered by firm and reported in the parenthesis.

***, ** and *represent significance at the 1%, 5%, and 10% levels, respectively.

### 3. The influence of the change of official performance assessment

The root of the phenomenon of top-down amplification of economic growth targets lies in the GDP-oriented officials’ promotion tournament [24]. However, with China’s economic development transformation, the criterion that the central government evaluates official performance is also changing. Especially after 2012, the indicators referring to GDP growth rate are gradually replaced by the indicators reflecting the innovation and environmental protection. We regard 2012 as the demarcation point of the reform of government officials’ performance appraisal, and set a dummy variable denoting as *Reform* (the value is 1 after 2012, otherwise the value is 0) and an interaction term denoting as *Constraints-Reform*. The regression results are shown in **Table 8**. The interaction coefficient of local governments’ economic growth target constraints and the reform of officials’ performance assessment is positively significant at least at the 5% level, indicating that the reform of government officials’ performance appraisal efficiently alleviates the negative impact of the economic growth target constraints on enterprise technological innovation, which supports H2.

**Table 8:** The influence of the change of official performance assessment.

	(1)	(2)	(3)	(4)	(5)	(6)
	R&D	R&D	R&D	Patent	Patent	Patent
**Constraint1**	-0.133***			-0.074***		
	(0.038)			(0.022)		
**Constraint1-Reform**	0.078**			0.062***		
	(0.039)			(0.023)		
**Constraint2**		-0.046			-0.049***	
		(0.035)			(0.016)	
**Constraint2-Reform**		0.087**			0.056**	
		(0.038)			(0.023)	
**Constraint3**			-0.075***			-0.052***
			(0.027)			(0.012)
**Constraint3-Reform**			0.063**			0.049***
			(0.029)			(0.015)
**Size**	0.686***	0.723***	0.720***	0.510***	0.529***	0.528***
	(0.033)	(0.037)	(0.036)	(0.036)	(0.038)	(0.037)
**Tobin Q**	0.047***	0.037**	0.037**	0.066***	0.068***	0.070***
	(0.013)	(0.015)	(0.015)	(0.013)	(0.013)	(0.013)
**Lev**	-0.447***	-0.450***	-0.442***	-0.259*	-0.323**	-0.330**
	(0.140)	(0.165)	(0.162)	(0.140)	(0.148)	(0.145)
**Age**	-0.025***	-0.028***	-0.028***	-0.014***	-0.017***	-0.017***
	(0.005)	(0.006)	(0.006)	(0.005)	(0.005)	(0.005)
**Top5**	0.000	0.000	-0.000	-0.006***	-0.007***	-0.007***
	(0.002)	(0.002)	(0.002)	(0.002)	(0.002)	(0.002)
**ROA**	1.731***	2.397***	2.368***	1.553***	1.578***	1.594***
	(0.317)	(0.398)	(0.393)	(0.409)	(0.428)	(0.422)
**Staff**	0.326***	0.259***	0.257***	0.087***	0.075***	0.074***
	(0.023)	(0.022)	(0.022)	(0.017)	(0.017)	(0.017)
**Dum_state**	-0.063	-0.068	-0.060	0.074	0.076	0.089
	(0.068)	(0.073)	(0.073)	(0.060)	(0.062)	(0.062)
**Industry FE**	Yes	Yes	Yes	Yes	Yes	Yes
**Year FE**	Yes	Yes	Yes	Yes	Yes	Yes
**N**	14607	11338	11539	12221	11090	11293
**F**	249.736	180.042	188.545	43.351	42.152	43.137
**Adjusted R** ^**2**^	0.544	0.518	0.519	0.238	0.244	0.245

Notes: The standard errors are clustered by firm and reported in the parenthesis.

***, ** and *represent significance at the 1%, 5%, and 10% levels, respectively.

## V. Further discussion

### 1. Heterogeneity check

The above has conducted a benchmark analysis of the relationship between economic growth target constraints and enterprise technological innovation. Considering that ownership, industry characteristics, and market environment of the enterprise are different, the response of enterprises to government behavior may also be different. For this, we use *Constraint3*, which is the comprehensive result of multi-level government competition, to measure the local government’s economic growth target constraints and investigate their heterogeneous impact on enterprise technological innovation.

#### 1.1 Ownership differences

Referring to the previous literature [42], we divide the sample into two subsamples of state-owned enterprises (SOEs) and non-state-owned enterprises (NSOEs). Column (1) of **Table 9** provides the regression results of the state-owned enterprise sample, and Column (2) provides the regression results of the non-state-owned enterprise sample. It can be seen that the negative effect of the government’s economic growth target constraints is more evident in the sample of SOEs. In China, local governments have strong control over SOEs’ production and operation activities. When the government faces issues such as adjustments to economic development strategies, fiscal deficit, or promotion of government officials, the government will transfer some of the policy burdens and economic pressure to SOEs and encourage SOEs to invest in the light of their willings and preferences. At the same time, it is the mission of SOEs to respond to the government’s appeal actively [34]. Therefore, when the local governments face tremendous pressure in the economy growth, SOEs tend to invest in projects with a short investment cycle and quick return to meet the needs of the government and boost the regional economy, leading to a significant decline in enterprise technological innovation.

**Table 9:** Heterogeneity check.

	(1)	(2)	(3)	(4)	(5)	(6)
	SOEs	NSOEs	Regulated industries	Non-Regulated industries	Higher marketization	Lower marketization
**Constraint3**	-0.049**	-0.023*	-0.054***	-0.017	0.030	-0.044***
	(0.020)	(0.014)	(0.019)	(0.014)	(0.037)	(0.013)
**Size**	0.566***	0.502***	0.521***	0.531***	0.537***	0.528***
	(0.055)	(0.046)	(0.064)	(0.045)	(0.061)	(0.045)
**Tobin Q**	0.055*	0.070***	0.115***	0.046***	0.032	0.082***
	(0.029)	(0.015)	(0.023)	(0.015)	(0.025)	(0.015)
**Lev**	-0.994***	-0.009	-0.677**	-0.053	-0.365	-0.331*
	(0.270)	(0.159)	(0.265)	(0.163)	(0.237)	(0.182)
**Age**	-0.018	-0.018***	-0.032***	-0.008	-0.006	-0.020***
	(0.013)	(0.005)	(0.010)	(0.006)	(0.010)	(0.006)
**Top5**	-0.009***	-0.006***	-0.010***	-0.005***	-0.004	-0.008***
	(0.003)	(0.002)	(0.003)	(0.002)	(0.003)	(0.002)
**ROA**	1.580**	1.424***	1.843**	1.894***	1.994***	1.519***
	(0.722)	(0.486)	(0.717)	(0.480)	(0.768)	(0.501)
**Staff**	0.047*	0.098***	0.061**	0.076***	0.038	0.084***
	(0.026)	(0.022)	(0.031)	(0.020)	(0.033)	(0.020)
**Dum_state**			0.316***	-0.040	0.065	0.105
			(0.115)	(0.069)	(0.145)	(0.069)
**Industry FE**	Yes	Yes	Yes	Yes	Yes	Yes
**Year FE**	Yes	Yes	Yes	Yes	Yes	Yes
**N**	3564	6641	3354	6851	2760	7443
**F**	21.382	31.092	17.182	33.506	15.851	32.025
**Adjusted R** ^**2**^	0.322	0.195	0.281	0.238	0.234	0.252

Notes: The dependent variable is *Patent*. The standard errors are clustered by firm and reported in the parenthesis.

***, ** and *represent significance at the 1%, 5%, and 10% levels, respectively.

#### 1.2 Industry characteristics

Since different industries differ in capital structure, degree of competition, and dependence on policies [43, 44], the impact of the governments’ economic growth target constraints on enterprise technological innovation may be different at the industry level. Generally speaking, compared with non-regulated industries, regulated industries have a higher dependence on policies. Meanwhile, more policy restrictions in regulated industries. Referring to [40], we divide the sample into two sub-samples of regulated and non-regulated industries. According to the Guidance on Industry Classification of Listed Companies issued by the Certification Association, mining industry, chemical fiber manufacturing, the production and supply industry of electricity, heat, gas and water, transportation industry, and storage industry are defined as regulated industries. The regression results are shown in Column (3) and Column (4) of **Table 9**, respectively. In the regulated industries, the estimated coefficient of *Constraint3* is negatively significant at the 1% level, while the estimated results of non-regulated industries are not significant, supporting the above reference.

#### 1.3 Regional marketization level

At present, China is in the process of marketization, but because of the differences in regional resources, policies, and governance levels, regional marketization exists significant differences [44]. In areas with low marketization levels, local governments intervene more in resource allocation, making enterprises in the jurisdiction have to bear many political targets, leading to the lack of independence in decision-making for enterprises and the insufficiency of enterprise technological innovation. At the same time, the imperfect market system will also cause that enterprises have difficulties in financing and protecting rights, which frustrate the enthusiasm for enterprise technological innovation. According to the *marketization index of China’s provinces*: *Neri report 2021*, we divide the sample into two subsamples of higher and lower marketization levels. During the sample period, the province whose marketization indicators are higher than the national average is defined as the region with a high marketization level. Conversely, it is defined as a region with a low marketization level. Column (5) and Column (6) of **Table 9** report the regression results. In the areas with lower marketization levels, the negative effect of economic growth target constraints on the enterprise technological innovation is more obvious.

### 2. Mechanism test

We have analyzed the relationship between local governments’ economic growth target constraints and enterprise innovation. Next, by using the way of mediation effect test [45], the influence mechanism of the government’s economic growth target constraints on enterprise technological innovation will be discussed from two aspects of financing constraints and the contribution of R&D investment to enterprise performance.

#### 2.1. Financing constraints

Under the drive of the economic growth target constraints, in order to flourish the economy of jurisdiction, local governments will attempt to intervene and control the allocation of land, capital, and other factors, resulting in the distortion in the price of factor market [25, 37], which in turn raises the enterprise financing cost and reduce enterprise technological innovation enthusiasm. Following the previous literature [46, 47], we use the SA index (*SA* = -0.043*size*size +0.737*size—0.04*age) to examine the mediating effect of financing constraints. The regression results are shown in Column (1) to Column (2) of **Table 10**. It can be observed that the estimated coefficient of the economic growth target constraints is negatively significant at the level of 1% when the dependent variable is *SA*. The SA index (*SA*) has a significant positive impact on the enterprise technological innovation. That is to say, the stronger the economic growth target constraints faced by local governments, the enterprise financing constraints will increase accordingly, which is not conducive to enterprise technological innovation. As indirect evidence, we also use government subsidy (*Subsidy* is defined as government subsidies obtained by the enterprises divided by operating revenue) as a mediator variable to examine, and the regression results are shown in Column (3) to Column (4) of **Table 10**. Government subsidies (*Subsidy*) have significantly promoted enterprise technological innovation, but economic growth target constraints have reduced government subsidies, which in turn hinders enterprise technological innovation.

**Table 10:** The results of the mechanism test.

	(1)	(2)	(3)	(4)	(5)	(6)
	SA	Patent	Subsidy	Patent	Contri	Patent
**Constraint3**	-0.040***	-0.031***	-0.002**	-0.031***	-0.019**	-0.032***
	(0.014)	(0.012)	(0.001)	(0.012)	(0.010)	(0.012)
**SA**		0.435***				
		(0.031)				
**Subsidy**				0.852***		
				(0.155)		
**Contri**						0.039**
						(0.017)
**Size**			-0.049***	0.569***	0.006	0.527***
			(0.002)	(0.037)	(0.021)	(0.037)
**Tobin Q**	-0.262***	0.063***	0.027***	0.049***	0.122***	0.066***
	(0.013)	(0.013)	(0.001)	(0.013)	(0.013)	(0.013)
**Lev**	2.684***	-0.300**	0.024**	-0.355**	-6.550	-0.067
	-0.262***	0.063***	0.027***	0.049***	0.122***	0.066***
**Age**			-0.000	-0.017***	-0.004	-0.017***
			(0.000)	(0.005)	(0.004)	(0.005)
**Top5**	0.013***	-0.008***	-0.001***	-0.006***	0.003**	-0.007***
	(0.002)	(0.002)	(0.000)	(0.002)	(0.001)	(0.002)
**ROA**	4.809***	1.785***	0.311***	1.242***	-0.553**	1.609***
	(0.297)	(0.417)	(0.026)	(0.413)	(0.273)	(0.422)
**Staff**	0.160***	0.074***	0.004***	0.070***	-0.031**	0.075***
	(0.019)	(0.017)	(0.001)	(0.017)	(0.013)	(0.017)
**Dum_state**	0.448***	0.092	0.003	0.088		0.092
	(0.060)	(0.060)	(0.004)	(0.061)		(0.062)
**Industry FE**	Yes	Yes	Yes	Yes	Yes	Yes
**Year FE**	Yes	Yes	Yes	Yes	Yes	Yes
**N**	13488	10205	13488	10205	13487	10205
**F**	167.229	47.576	297.681	43.551	469.822	39.531
**Adjusted R** ^**2**^	0.488	0.245	0.478	0.249	0.657	0.245

Notes: The standard errors are clustered by firm and reported in the parenthesis.

***, ** and *represent significance at the 1%, 5%, and 10% levels, respectively.

#### 2.2. The contribution of R&D investment to enterprise performance

The purpose of enterprise technological innovation is to improve competitiveness and income. The more revenue obtained from the new products, the more R&D investment enterprises are willing to provide, and vice versa. Yu and Zhang [48] demonstrated that to accomplish the established economic growth target as soon as possible, local governments vigorously develop the real estate industry, leading to urban housing price rise and bringing considerable margins to the relative industry. Attracted by the high margins, enterprises may prefer to invest in the real estate sector and decrease the technological innovation investment. On this view, we test whether the local government’s economic growth target constraints can affect enterprise technological innovation by influencing the contribution of R&D investment to enterprise performance (*Contri*). Referring to Wang and Wen [44], *Contri* is defined as the enterprise revenue growth rate divided by the patent application. The results are shown in Column (5) to Column (6) of **Table 10**. It can be seen that the estimated coefficient of the economic growth target constraints is significantly negative at the level of 1%, indicating that the local government’s economic growth target constraints decreased the contribution of R&D investment to enterprise performance (*Contri*). The contribution of R&D investment to enterprise performance (*Contri*) implies the expectation of the enterprise for the innovative income and has a positive impact on the enterprise innovation activities, thus the regression coefficient of the *Contri* is positive. That is to say, local government’s economic growth target constraints will inhibit enterprise technological innovation activities by decreasing the contribution of R&D investment to enterprise performance.

## VI. Conclusions

Under the administrative system of multi-level government and the promotion tournament of GDP-oriented, superior governments’ officials try their best to obtain beneficial element resources for economic growth to master the advantages of promotion and organize a political competition around economic growth among the subordinate government’s officials. When the economic growth target is proposed by the superior government, it will increase gradually along with the different levels of subordinate governments. The competition among multi-level government amplifies the incentive effect of local governments to pursue economic growth, exposing governments to economic growth target constraints, which profoundly affects the behavior of local governments, the allocation of the regional resources, and that in turn affects the production and operation activities of the enterprises under the jurisdiction through the “visible hand” of the local government.

Using the data from Chinese listed companies in the Shanghai and Shen Zhen A-share market for the period of 2007 to 2019 and the *Report on the Work of the Government*, we analyze the impact of economic growth target constraints on enterprise technological innovation. Our empirical results reveal that the local government’s economic growth target constraints have significantly negative effect on enterprise technological innovation. On average, with the economic growth targets of municipal governments are 1% higher than the central government, enterprise R&D expenditure and the invention patent applications will decrease by about 4.3% and 3.3%. As the government’s pursuit of economic growth target comes from the official promotion evaluation system, so the change of official performance evaluation indicators from the quantity to quality of economic growth can alleviate the negative effect of the local government’s economic growth target constraints on enterprise technological innovation. Heterogeneity analysis demonstrates that the restraining effect of the local government’s economic growth target constraints on enterprise technological innovation appears more obvious in the samples of SOEs, regulated industries, and the low marketization level. The Mechanism effect test indicates that local government’s economic growth target constraints will inhibit enterprise technological innovation by aggravating enterprise financing constraints and decreasing the contribution of R&D investment to enterprise performance.

Compared with the previous literature, we illustrate how the local government’s economic growth target constraints come into being under the multi-level government competition and test its impact and mechanism on enterprise technological innovation. Our study not only extends the research subject to the management of economic growth targets and enterprise technological innovation but also provides new empirical evidence for understanding the economic results of the local government’s behavior.

This paper has important implications for policymakers to optimize the official appraisal system and accelerate the establishment of the enterprise-dominated innovation system. Firstly, local governments should combine regional development and resource endowments to set the reasonable economic growth target, avoiding the adverse results of regional resource misallocation caused by blind competition. Secondly, it is necessary to optimize the official performance appraisal system, reduce the weight of GDP growth speed, increase the weight of scientific and technological innovation and environmental protection, which can reflect the economic development quality, and transform China’s institutional advantages into effective state governance. Thirdly, the local government’s short-term behavior under the pressure of economic growth targets will occupy abundant fiscal and credit resources, increase enterprise financing constraints, decrease the contribution of R&D investment to enterprise performance, and thus inhibit enterprise technological innovation. It is necessary to clarify the boundary between the government and the market, promote the transformation from local governments to service-oriented governments, reduce improper intervention in the economy, and create a good external environment for enterprise technological innovation. Finally, the impact of the local government’s economic growth target constraints on enterprise technological innovation exists heterogeneity. Therefore, to improve policy implementation efficiency, local governments should consider the industries and enterprise characteristics when making the relevant industrial policies.

## Supporting information

S1 Dataset Dataset used in this study. (DTA)
